# Superficial Tale of Two Functional Groups: On the Surface Propensity of Aqueous Carboxylic Acids, Alkyl Amines, and Amino Acids

**DOI:** 10.1021/acs.accounts.2c00494

**Published:** 2022-11-23

**Authors:** Olle Björneholm, Gunnar Öhrwall, Arnaldo Naves de Brito, Hans Ågren, Vincenzo Carravetta

**Affiliations:** † Division of X-ray Photon Science, Department of Physics and Astronomy, Uppsala University, Box 516, 75120 Uppsala, Sweden; ‡ MAX IV Laboratory, Lund University, Box 118, SE-22100 Lund, Sweden; § Department of Applied Physics, Institute of Physics “Gleb Wataghin”, Campinas University, CEP, 13083859 Campinas SP, Brazil; ∥ CNR-IPCF, Institute of Chemical Physical Processes, via G. Moruzzi 1, I-56124 Pisa, Italy

## Abstract

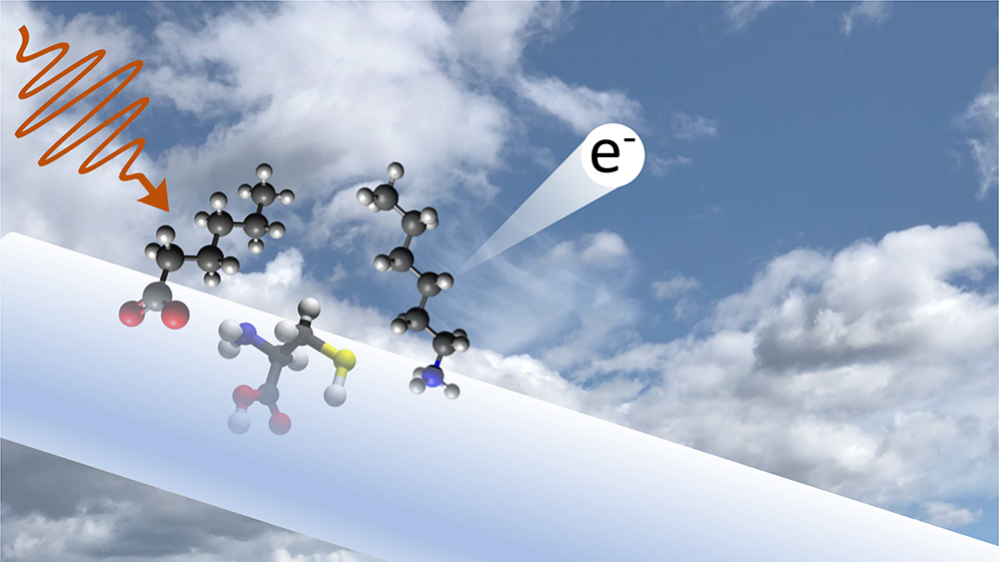

The gas–liquid interface of water is environmentally relevant due to the abundance of aqueous aerosol particles in the atmosphere. Aqueous aerosols often contain a significant fraction of organics. As aerosol particles are small, surface effects are substantial but not yet well understood. One starting point for studying the surface of aerosols is to investigate the surface of aqueous solutions. We review here studies of the surface composition of aqueous solutions using liquid-jet photoelectron spectroscopy in combination with theoretical simulations. Our focus is on model systems containing two functional groups, the carboxylic group and the amine group, which are both common in atmospheric organics. For alkanoic carboxylic acids and alkyl amines, we find that the surface propensity of such amphiphiles can be considered to be a balance between the hydrophilic interactions of the functional group and the hydrophobic interactions of the alkyl chain. For the same chain length, the neutral alkyl amine has a lower surface propensity than the neutral alkanoic carboxylic acid, whereas the surface propensity of the corresponding alkyl ammonium ion is higher than that of the alkanoic carboxylate ion. This different propensity leads to a pH-dependent surface composition which differs from the bulk, with the neutral forms having a much higher surface propensity than the charged ones. In aerosols, alkanoic carboxylic acids and alkyl amines are often found together. For such mixed systems, we find that the oppositely charged molecular ions form ion pairs at the surface. This cooperative behavior leads to a more organic-rich and hydrophobic surface than would be expected in a wide, environmentally relevant pH range. Amino acids contain a carboxylic and an amine group, and amino acids of biological origin are found in aerosols. Depending on the side group, we observe surface propensity ranging from surface-depleted to enriched by a factor of 10. Cysteine contains one more titratable group, which makes it exhibit more complex behavior, with some protonation states found only at the surface and not in the bulk. Moreover, the presence of molecular ions at the surface is seen to affect the distribution of inorganic ions. As the charge of the molecular ions changes with protonation, the effects on the inorganic ions also exhibit a pH dependence. Our results show that for these systems the surface composition differs from the bulk and changes with pH and that the results obtained for single-component solutions may be modified by ion–ion interactions in the case of mixed solutions.

## Key References

- WernerJ.; PerssonI.; BjörneholmO.; KaweckiD.; SaakC.-M.; WalzM.-M.; EkholmV.; UngerI.; ValtlC.; CalemanC.; ÖhrwallG.; PrisleN.Shifted Equilibria of Organic Acids and Bases in the Aqueous Surface Region. Phys. Chem. Chem. Phys.2018, 20, 2328110.1039/C8CP01898G30191936PMC6146375.^1^ Liquid-jet X-ray photoelectron spectroscopy (XPS) measurements of the pH-dependent surface propensity of alkanoic carboxylic acids and alkyl amines.
- EkholmV.; CalemanC.; Bjärnhall PrytzN.; WalzM.-M.; WernerJ.; ÖhrwallG.; RubenssonJ.-E.; BjörneholmO.Strong Enrichment of Atmospherically Relevant Organic Ions at the Aqueous Interface: The Role of Ion Pairing and Cooperative EffectsPhys. Chem. Chem. Phys.2018, 20, 2718510.1039/C8CP04525A30339167.^2^ A liquid-jet XPS and molecular dynamics (MD) study of how alkanoic carboxylates acids and alkyl ammonium ions together have an increased surface propensity due to ion pairing.
- CarravettaV.; GomesA.; MontiS.; MocellinA.; MarinhoR.; BjörneholmO.; ÅgrenH.; de BritoA. N.pH-Dependent X-ray Photoelectron Chemical Shifts and Surface Distribution of Cysteine in Aqueous Solution. J. Phys. Chem. B2019, 123, 377610.1021/acs.jpcb.9b0086630964991.^3^ A liquid-jet XPS and MD study of how the surface speciation of cysteine differs from the bulk.

## Introduction

The gas–liquid interface of water, henceforth denoted as the water surface, is of immense environmental importance as the oceans cover 71% of the earth’s surface area, and aqueous particles, ranging in size from submicrometer aerosol particles to millimeter-sized raindrops, are abundant in the atmosphere. Atmospheric aerosols affect the global radiation balance, directly through the scattering of sunlight and indirectly as an important source of cloud condensation nuclei (CCN). The effects of aerosols have been identified by the UN Intergovernmental Panel on Climate Change (IPCC) as major uncertainties in climate models,^4^ and a better understanding of these is important for improving climate modeling. Atmospheric aerosols contain a wide variety of species, such as inorganic ions from the oceans; organic molecules from both direct emissions and the decomposition of biomaterial; soot from combustion, pollutants, and mineral particles; and a range of organic and inorganic species from the oxidation of precursor gases throughout the atmosphere. Depending on the environment, organic compounds constitute 20–90% of the submicrometer aerosol mass.^5^ These organics are mainly formed through gas-to-particle conversion resulting in so-called secondary organic aerosols (SOA) with a broad range of complex compositions. Due to the small size of aerosols, surface phenomena and processes become especially important. This includes surface-active species affecting surface tension, along with condensation and evaporation rates,^6^ and thus aerosol growth and CCN activity, as well as their chemical activity. Moreover, as small aerosols are microscopic systems, surface enrichment of a species may lead to bulk depletion, affecting macroscopic properties. These examples illustrate that important properties of aerosols are influenced by the composition and internal spatial distribution of chemical species. The atmospheric science community is now beginning to recognize the need to seriously account for surface effects in climate models,^7^ making it crucial to improve our molecular-level understanding of aerosol surfaces.

One starting point for this is to probe the surface of aqueous solutions using X-ray photoelectron spectroscopy (XPS) in combination with a liquid jet, which has been done for selected representative molecules in aqueous solutions. One type of system is liquid mixtures of water and more or less amphiphilic molecules with a hydrophilic and a hydrophobic part. We can consider the solvation of the amphiphile as a balance between the hydrophilic and hydrophobic interactions. For acetonitrile, alcohols, and carboxylic acids,^1,2,8−12^ XPS studies supported by molecular dynamics (MD) simulations have shown an increased surface enrichment of the amphiphile with increasing length of the hydrophobic alkyl chain, with the hydrophilic part solvated and the hydrophobic part to some degree desolvated. With increasing surface coverage, the hydrophobic parts undergo orientational changes from mainly lying down at low surface coverage to standing up to make room for more molecules with increasing coverage. See, for example, refs (7) and (10).

The presence of amphiphilic molecules at the surface may also affect the distribution of inorganic ions. For neutral molecules, butanol and butyric acid have been seen to have opposite effects on the abundance of bromide and iodide at the surface.^13^ Charged surfactants containing, for example, carboxylate or ammonium groups, have been shown to attract ions of opposite charge.^14,15^ As these functional groups are titratable, i.e., they can either lose or take up a proton depending on the pH, the effects on the inorganic ions have been shown to be pH-dependent.^14^ The surface composition generally differs from the bulk composition in terms of both speciation and concentration. Surface enrichment varies depending on the species and conditions, and concentrations 1 to 2 orders of magnitude higher have been reported for relatively small organic compounds.^9,16^ The carboxylic and amine groups are two common titratable functional groups in organics. Molecules containing carboxylic and amine groups, e.g., carboxylic acids, alkyl amines, and amino acids,^1−3,12,17−22^ can thus be expected to have surface propensities depending on both the nature of any hydrophobic groups and the pH-dependent protonation state of the functional group, which is the topic of this Account. Here we will review some results, obtained with liquid-jet XPS and theoretical simulations, of the surface behavior of molecules containing carboxylic and amine groups in both individual carboxylic acids and alkyl amines species, together in a mixed solution and combined into amino acids.

## Methods

To study the surface composition of aqueous solutions, liquid-jet XPS combining chemical selectivity and surface sensitivity has become an established tool. This has been extensively reviewed,^23−25^ and here we will only briefly mention some key points important to this review. In XPS, an incoming photon of energy *h*ν ionizes a core electron from one of the atoms in the sample. By measuring the kinetic energy (KE) of the emitted electrons using an electron spectrometer, one can obtain the sample-specific electron binding energy (BE) as *h*ν-KE. Two properties of XPS are especially important in the present context: its chemical selectivity and surface sensitivity. The chemical selectivity is due to the core electron BEs being characteristic for each element, thus providing information on sample composition. Moreover, the exact BE depends on the formal oxidation state of the atom and its local chemical and physical environment. This so-called chemical shift makes it possible to separate chemically inequivalent atoms of the same element. In the present context, C 1s XPS can separate between the carbon atoms in the alkyl chain and the carboxylic group of a carboxylic acid, as well as between its protonated and deprotonated forms −COOH/–COO^–^. Similarly, the probing of N 1s allows distinguishing between the amine and ammonium groups (−NH_2_/–NH_3_^+^). The surface sensitivity is due to the outgoing electrons having a high cross section for inelastic scattering, which means that the bulk signals are suppressed relative to the surface signals. At around 50–100 eV KE, the photoelectron inelastic mean free path has a minimum on the order of 0.5–1 nm.^26,27^ As the KE for electrons from a core level of a certain BE depends on the used *h*ν, the surface sensitivity can be varied by selecting an appropriate *h*ν. The short photoelectron inelastic mean free path also means that XPS requires a high vacuum for the photoelectrons to make it to the spectrometer and through it to the detector. The high vapor pressure prevented electron spectroscopy of aqueous systems for a long time until the introduction of the liquid jet, which in combination with differentially pumped “ambient pressure” electron spectrometers has become a powerful tool; see, for example, ref (28). In addition, the liquid jet implies continuous sample renewal, thus avoiding the problems of radiation damage and surface contamination. In summary, liquid-jet XPS combines chemical selectivity and surface sensitivity, both important aspects for the studies reviewed here.

The main observables in XPS are the intensities and BEs. The intensities depend on the amount and distribution relative to the surface of the species, while the BEs depend on the local environment of the atom. To interpret these observables in terms of the geometric and electronic structure of the system, comparisons with numerical simulations are extremely useful, especially in the case of complex systems such as solutions. These concern both the microscopic composition, structure, and dynamics of the sample and the prediction of the BEs for the different ionization sites. While calculations of BEs require quantum mechanical (QM) methods, the molecular dynamics is generally simulated using classical methods of molecular mechanics (MM) based on force fields describing the interactions between the involved species. Recently, combined QM-MM methods have become accessible, in which a small region containing a few molecules is treated with QM and a much larger surrounding region is treated classically but interacts with the first one. The need to resort totally or partially to MM is simply due to the large number of molecular components of the system, resulting in extreme computational costs for QM. This is evidently the case for aqueous solutions where, in order to have a statistically significant description of both composition and configurational sampling, the system can include tens of thousands of molecules. For an explanation of the various aspects of QM-MM applied to XPS and liquid solutions, see ref (29). These difficulties are even more accentuated in the case of simulations of liquid surfaces where the translational symmetry is reduced from three to two dimensions. The system has been described as a slab in the case of simulations of both the bulk and the surface of the solution. Most of the systems reviewed here consist of stable, well-defined species for which we used traditional force fields that include interactions such as Coulombic, van der Waals, and hydrogen bonding. For cysteine with three different protonation centers, however, we used a reactive force field to adequately describe the proton transfer both in the solute and in the solvent in aqueous solutions at different pH values. The reactive force field employed,^30^ which was previously optimized by quantum calculations for the description of amino acids,^31^ contains a large number of additional terms capable of describing the breaking and formation of chemical bonds in addition to the usual above-mentioned terms.

## Carboxylic Acids and Alkyl Amines

Carboxylic acids are common products of oxidation processes in nature and have motivated the large amount of interest in the surface properties of their binary aqueous solutions. A number of studies using liquid-jet XPS from our group and others have elucidated some of the complex dependencies of the chain length (degree of hydrophobicity), concentration, and pH (charge state of the functional group) on the surface structure of aqueous solutions of alkanoic carboxylic acids and their conjugate bases, the carboxylate salts.^1,12,17^

Generally, an amphiphilic molecule such as an alkanoic carboxylic acid is expected to increase its propensity to go to the surface the longer the aliphatic chain is, as the hydrophobicity of the chain outweighs the hydrophilicity of the carboxylic functional group; see, for example, ref (17). The alkanoic carboxylic acids are all surface-active in the sense that they lower the surface tension and exhibit a positive surface excess at the vapor/liquid interface, with a higher concentration at the surface than in the bulk. Their conjugate bases show a more diverse picture in that the smallest carboxylate ions, formate and acetate, avoid the surface, and the rest, from propionate and on, have a positive surface excess but a weaker affinity for the surface than the corresponding carboxylic acid, which can be understood by the larger hydration energy of the charged carboxylate −COO^–^ group compared to the carboxylic −COOH group. The surface structure is also strongly affected by the concentration of the solute: at low concentrations, the surface adsorption is unhindered and the concentration at the surface will increase linearly with the solute concentration, but competition for surface adsorption sites will increase with concentration until a monolayer of adsorbed molecules is formed.

**Figure 1:**
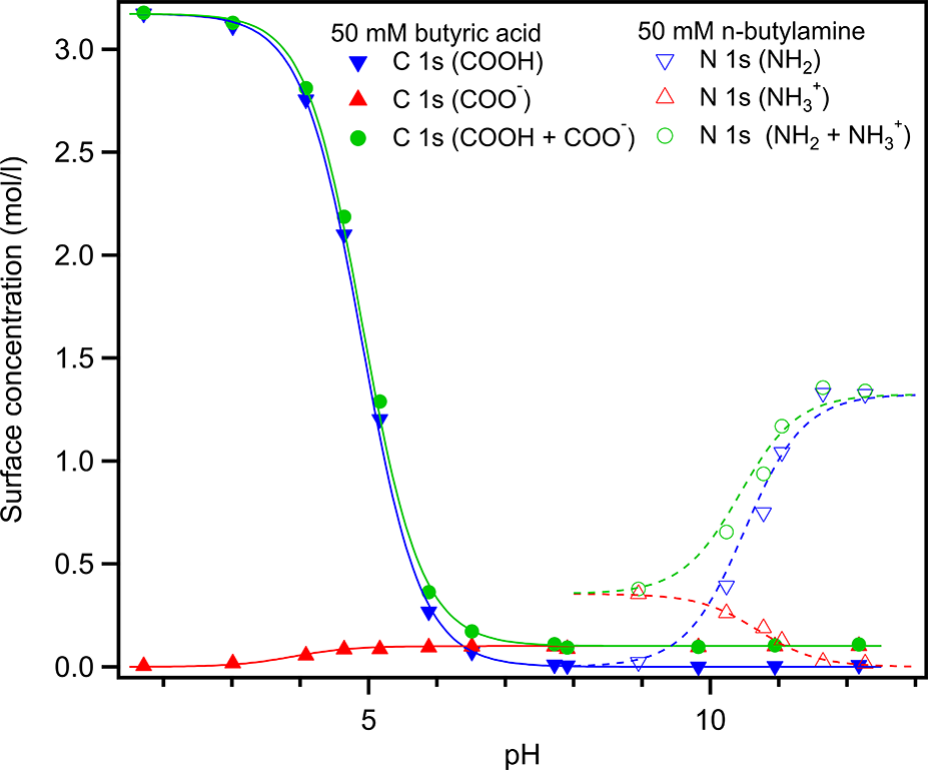
Estimated surface concentration of 50 mM butyric acid in water (solid lines and filled symbols) and 50 mM *n*-butylamine (dashed lines and empty symbols) from the intensities of the C 1s line of the carboxylic carbon in butyric acid and the N 1s line in butylamine, recorded using surface-sensitive liquid-jet XPS.

Due to the different surface propensities of neutral and charged forms, the neutral form is more prevalent at the surface than the known equilibria for the speciation of carboxylic acids (described by the Henderson–Hasselbalch equation) would indicate. It would thus appear that the acid dissociation constant *K*_a_ is larger at the surface than in the bulk, but we have developed a model that in addition to the speciation as a function of pH also takes the different surface propensities into account, which describes the observed phenomenon well.^1^ Also for the alkyl amines the neutral form has a larger surface propensity than the corresponding charged protonated form. However, for the same alkyl chain length, the neutral alkyl amine (dominating at high pH) is less prone to adsorb at the surface than the neutral alkanoic carboxylic acid (dominating at low pH), whereas the surface propensity of the conjugate acid of the amine (dominating at low pH) is higher than that of the conjugate base of the carboxylic acid (dominating at high pH).^10^ This is illustrated in Figure 1, which shows the surface concentrations estimated from XPS spectra of aqueous solutions of butyric acid and *n*-butylamine using a simple two-layer model.^1^ Clearly, the surface propensity of the carboxylate ion is much lower than that of the ammonium ion, whereas the reverse is true for the neutral carboxylic acid and amine, indicating corresponding differences in hydration.^32^

In atmospheric aerosols and other naturally occurring aqueous systems, many types of chemical species typically occur together. A step toward understanding the surface properties of such complex systems is to study the surface propensity of a carboxylic acid and an alkyl amine together. The results above for carboxylic acids and alkyl amines separately show that the surface propensity is higher for the neutral forms than for the charged forms. This means that the carboxylic acids will have a higher surface propensity at low pH, whereas alkyl amines will have a higher surface propensity at high pH; see Figure 1. Consider a solution of equal concentrations of a carboxylic acid and an alkyl amine. The individual surface propensities suggest relatively reduced amounts of organics in the form of carboxylate and alkyl ammonium ions at the surface at the most environmentally relevant pH values. In order to assess this predicted behavior, surface-sensitive C 1s XPS spectra were compared between solutions containing sodium hexanoate and hexyl ammonium chloride separately and mixed in the intermediate pH range where both species are in their charged forms.^2^ The C 1s intensity of the mixed solution *I*(50 mM hexanoate + 50 mM hexyl ammonium) was 3 to 4 times higher than the sum of the C 1s intensities of the individual solutions, *I*(50 mM hexanoate) + *I*(50 mM hexyl ammonium). The observed C 1s intensity depends on contributions from both the bulk and the surface. As the bulk concentration is equal for all solutions, the difference is due to the surface. The higher intensity observed for the mixed solution implies a higher surface propensity of both components in the mixed solution compared to that in the separate solutions.

**Figure 2:**
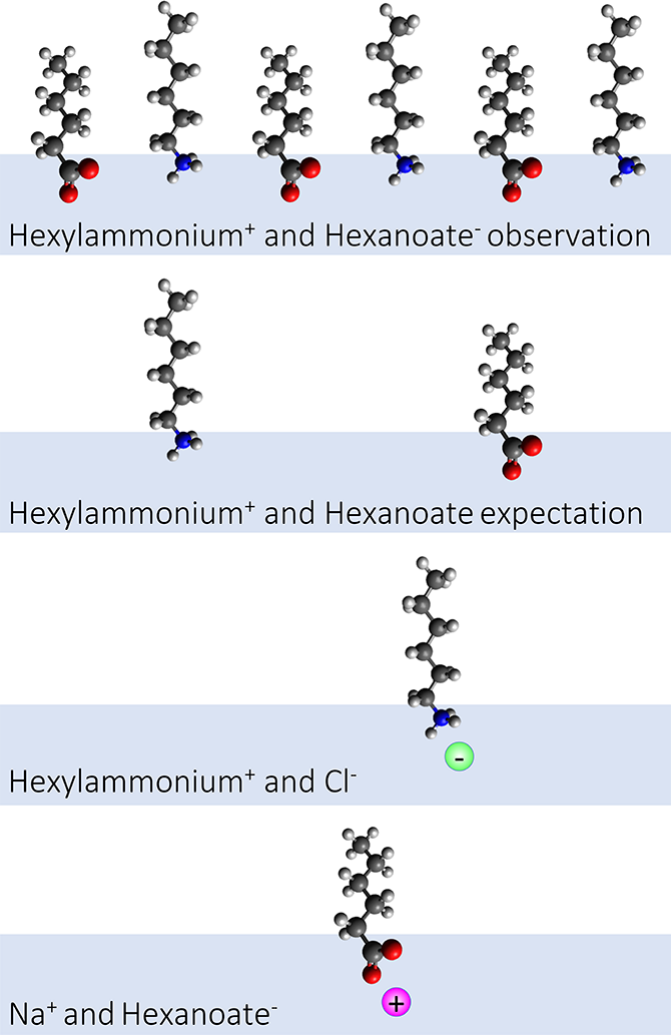
Schematic illustration of the surface composition aqueous solutions of a carboxylate ion and an alkylammonium ion, i.e., their environmentally most relevant forms, alone with inorganic counterions and together. The number of molecular ions in the figure schematically illustrates the relative numbers of molecular ions at the solution surface.

To understand why the behavior of the mixed system differs from the prediction based on the behavior of the individual components, we have to consider the role of the counterions; see Figure 2. In the solutions of the individual components, the counterions are Na^+^ for hexanoate and Cl^–^ for hexyl ammonium. Both Na^+^ and Cl^–^ are small inorganic ions with low surface propensity. For the solutions of the individual components, MD simulations indicate the organic ions to be enriched at the surface with their alkyl chains outside, the charged −COO^–^/–NH_3_^+^ groups solvated in the surface, and the Na^+^/Cl^–^ counterions solvated in the bulk, with a small increase in the outermost bulk layer. This layered structure of the cations and anions at the surface is reminiscent of the surface structure of inorganic salt solutions, as reviewed in ref (33), and can be considered to be an electric double layer in between the water bulk and the alkyl chains outside the solution. The mixed solutions contain both positively and negatively charged organic ions. The HexNH_3_^+^ N 1s binding energy is 0.36 eV lower for the mixed case than for the unmixed case, which is qualitatively compatible with the effects of a nearby negative charge such as the −COO^–^ group. This indicates ion pairing between the charged −COO^–^ and −NH_3_^+^ groups solvated in the surface layer. The MD simulations suggest the formation of larger entities of contact ion pairs in the form of zigzag chains composed of alternating positive and negative ions at the surface.

Some simple arguments are useful for a qualitative understanding of the different driving forces behind the different structural motifs of the pure and mixed cases. In the pure case with an outer layer of organic ions and an inner layer of inorganic ions, it is unfavorable to pull up inorganic ions from the bulk, and there will be Coulomb repulsion between equally charged ions within both layers. In the mixed case with an outer layer of alternating positively and negatively charged organic ions, Coulomb interaction is favorable and the oppositely charged ions act as counterions for each other. The surface propensity of the organic ions is enhanced by the cooperative effect of ion pairing.

In summary, we see that cooperative behavior on the microscopic scale leads to a more organic-rich and hydrophobic surface than would be expected from the bulk composition at environmentally relevant pH values. This illustrates that results obtained for single-component solutions may be modified by ion–ion interactions in the case of mixed solutions.

## Amino Acids

Amino acids combine the carboxylic and amine functional groups into biomolecules, which are important building blocks of all life forms on earth. Their structure can be described as H_2_N–CRH–COOH, where R stands for a side group that distinguishes the different amino acids. Studying them by liquid-jet XPS allows us to probe their properties at the liquid–vacuum interface. Amino acids of primarily biogenic origin are atmospherically relevant due to their CCN activity^34^ and are the most abundant water-soluble organic nitrogen-containing compounds in atmospheric aerosols.^35^ Amino acids combine the pH dependence of the carboxylic group and an amine group. At low pH, they are found in a cationic form, ^+^H_3_N–CRH–COOH, and at high pH, they are found in an anionic form, H_2_N–CRH–COO^–^. At intermediate pH, the amino acids occur in a zwitterionic form, ^+^H_3_N–CRH–COO^–^, which is overall neutral but contains two oppositely charged groups.

From a fundamental point of view, we could ask how the presence of the carboxylic and amine groups in a single molecule affects the amino acid surface propensity compared to that of their simpler counterparts, carboxylic acids and alkyl amines. In addition, how do their side groups R, such as chains and rings, alter their surface propensity? The amino acid cysteine also has a third titratable sulfur group, which raises the question of how the deprotonation of this group affects the surface propensity compared to (de)protonation of the carboxylate or amine groups? As discussed above, the pH significantly changes the surface propensity of both carboxylic acids and alkyl amines.^1,2^ This fact leads to another issue of how the amino acid surface propensity changes with pH. A more realistic system is an aqueous solution with salt and amino acid. Common salts containing chloride tend to avoid the solution interface, which leads to the issue of how their interaction with amino acids affects their surface propensity.

Glycine with R = H is the simplest amino acid, which makes it a natural starting point for addressing in a stepwise manner the questions posed above. The effects of protonation/deprotonation on the BE of the two C 1s glycine levels and one N 1s glycine level have been studied in detail.^36^ For example, we observe a 2.5 eV shift of the N 1s BE between the −NH_2_ and −NH_3_^+^ forms. The various contributions of protonation/deprotonation and solvent-induced screening to the chemical shifts could be established by supporting calculations. The C 1s and N 1s XPS BEs thus serve as fingerprints of the pH-dependent protonation states of the amino acid.

**Figure 3:**
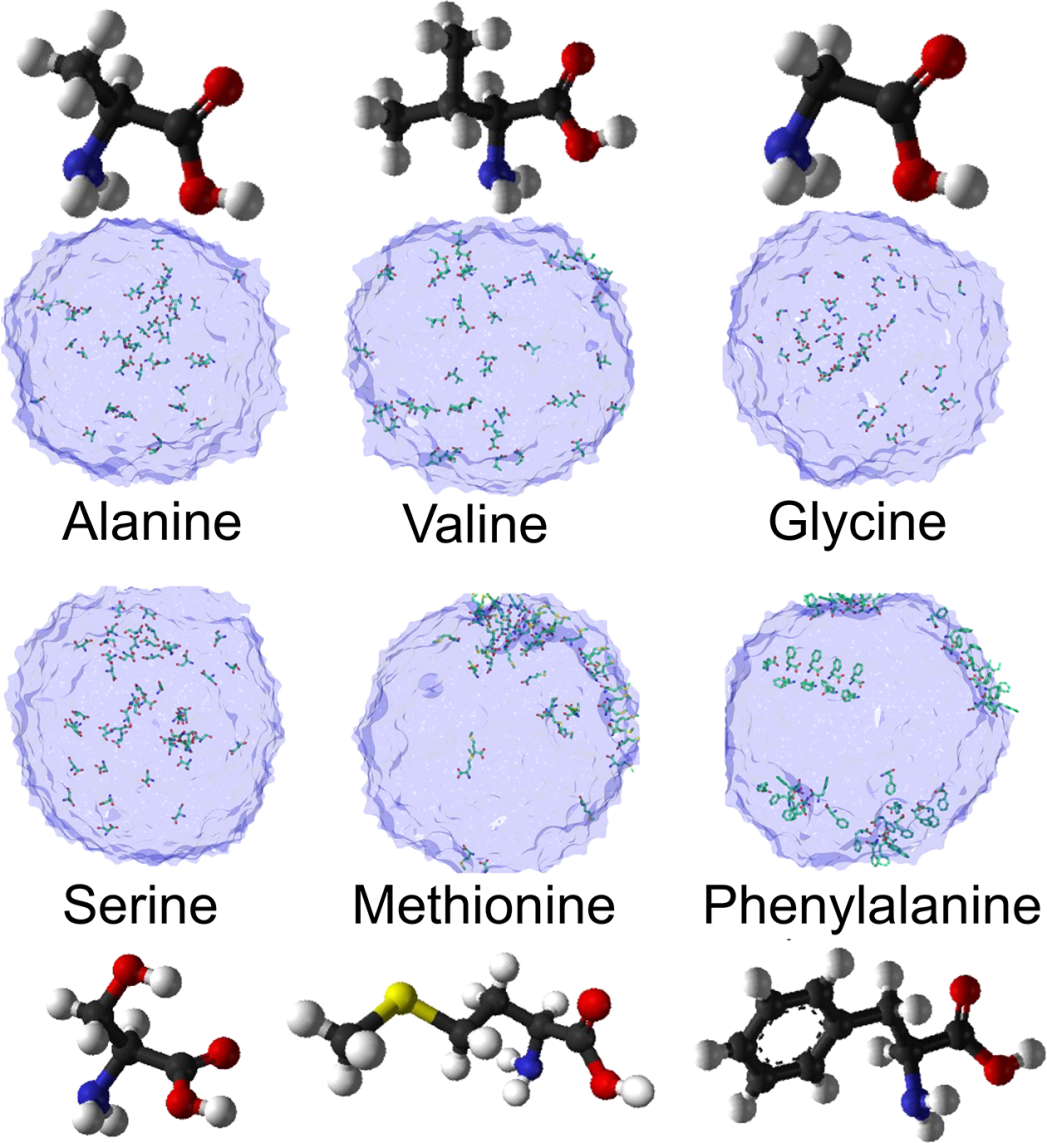
Cross section of small aqueous droplets with selected amino acids from MD simulations, showing the distribution between the bulk and surface. Original data from ref (37).

How does the nature of the side group R affect the amino acid surface propensity? Using MD, we have studied the surface propensity in small aqueous droplets of some amino acids, which have been found in aerosols and rain.^37^ Serine, glycine, and alanine were predicted to stay in the bulk (Figure 3) due to their strong hydrophilicity arising from the zwitterionic backbone and a smaller side group, while valine, methionine, and phenylalanine concentrate on the surface owing to an amphiphilic effect dependent on their bulky and nonpolar side groups. In a separate XPS study,^22^ we determined the surface concentration for some amino acids in their zwitterionic form using a simple model in which glycine was used as a reference and was assumed to have no surface presence. The resulting surface concentration divided by the bulk concentration was found to be 0.9 for alanine, 7.6 for methionine, and 9.8 for valine. Qualitatively, we can see that the surface propensity increases with the size of the aliphatic side chain from glycine over alanine to valine, corroborating the earlier MD study.^37^ The surface propensity of methionine is lower than that of valine, despite the side group being larger for methionine, which can be understood as being due to the difference in the internal structure of the side groups. While the valine side group is purely hydrophobic, the presence of sulfur in the methionine side group enables some hydrogen bonds with water. This leads to a reduced hydrophobicity of the side group and consequently to a reduced surface propensity relative to valine. These results may be connected to the observation that the amino acids most often detected in aerosols have polar or charged side chains and hence a relatively lower surface propensity while the least abundant ones have hydrophobic side groups and hence a relatively higher surface propensity.^38^ The observed lower abundance of amino acids with hydrophobic side groups could be due to these being more surface-enriched, thereby becoming more available for oxidation into smaller and more volatile molecules.^37^ Supporting this oxidation hypothesis, laboratory experiments show the presence of l-methionine oxidation products.^39^ Furthermore, another study shows that dissolved amino acids including l-methionine can react with hydroxyl radicals in the atmosphere.^40^

Another sulfur-containing amino acid is cysteine (R = CH_2_SH), for which the −SH group is also titratable. With three titratable sites, cysteine can exist in eight possible protonation states, with the distribution of these species depending in a complex way on the solution pH. We investigated the pH-dependent cysteine surface propensity in refs (3) and (41). The ratio between the total signal intensities at pH = 1 and 5.0 is 1.7 (1.6) for the carbons (nitrogen). The only change in protonation between these two pH values is the deprotonation of the COOH group into COO^–^. In other words, COOH deprotonation causes a reduction in the surface propensity of cysteine by ∼1.6, i.e., behavior that is qualitatively similar to that of the carboxylic acids discussed above. The effect of −SH deprotonation into −S^–^ and −NH_3_^+^ into NH_2_ can be monitored at pH 9.5, where all of these species are present. Varying the XPS information depth between ∼1.2 and ∼3.2 nm by measuring at different photon energies,^26^ we could see that the deprotonation of the sulfur gives rise to an ∼1.7-fold reduction in the surface presence, while nitrogen deprotonation gives rise to an increase of ∼2.2. This second result is more complex to analyze because it assumes that the effects of the other two protonatable groups average out. Moreover, the surface-sensitive measurements are not compatible with the well-known bulk speciation.

**Figure 4:**
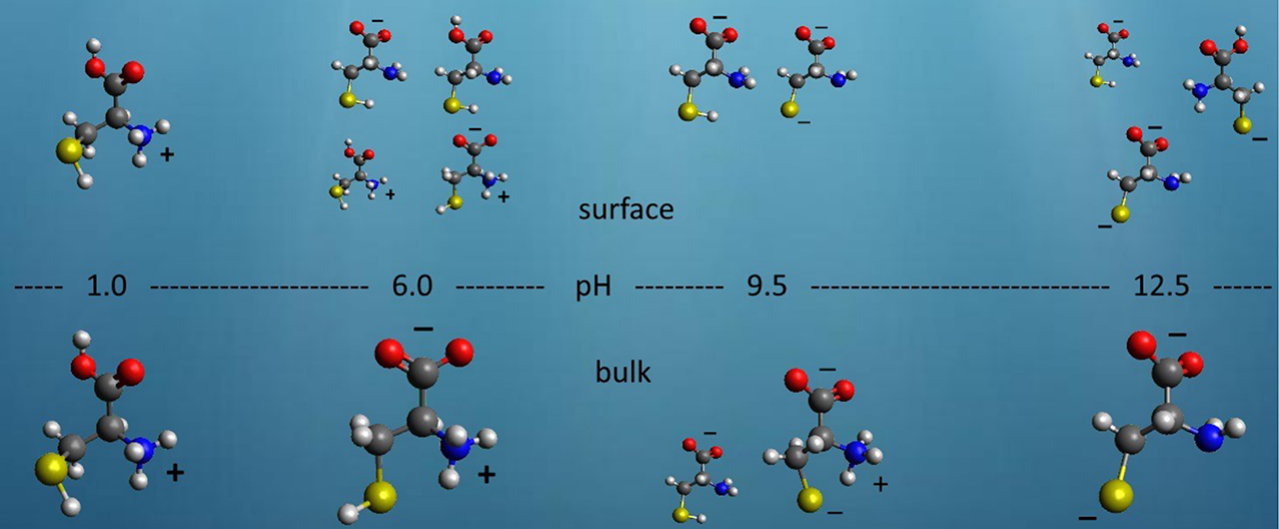
Most relevant species of cysteine in the bulk (bottom) and in the surface (top) as a function of the pH of the aqueous solution. The atoms of the structures are colored dark gray (carbon), red (oxygen), blue (nitrogen), yellow (sulfur), light gray (hydrogen), purple (Na), and green (Cl), where the last two are counterions. The apparent size of each structure is proportional to the relative abundance of the species; see also Table 1.

To obtain a better understanding of the cysteine surface speciation and propensity, we carried out simulations of the spatial distribution and XPS BEs of different species using reactive force field MM and ab initio QM calculations of BEs. These simulations reveal a cysteine speciation that is quite different at the surface compared to in the bulk at certain pH values. In particular, the appearance in the surface region of several species containing the −COOH group at pH ≥6 that are not present in the bulk is quite unexpected. This should not be taken as an indication of a high surface propensity for H^+^. The theoretical modeling and the XPS results together show that the bulk and the surface of the aqueous cysteine solution have quite different compositions, as schematically illustrated in Figure 4 and listed in Table 1. Another interesting result from the evaluation of the total XPS C 1s normalized area in this study in the present context is that at pH 9.5 the surface presence of cysteine is increased relative to that at pH 12.5 and 5.2. A plausible explanation of this observation is ion pairing, which is possible only at pH 9.5 according to the available surface species revealed by the XPS data.

**Table 1:** Relative Speciation of Cysteine in the Bulk and on the Surface Predicted by Reactive Molecular Dynamics on Model Systems Simulating an Aqueous Solution at Different pH Values^a^^,^^b^

pH region	NH_2_ S COOH	NH_2_ SH COO^–^	NH_2_ S^–^ COO^–^	NH_2_ SH COOH	NH_3_^+^ SH COOH	NH_3_^+^ SH COO^–^	NH_3_^+^ S^–^ COO^–^	NH_3_^+^ S^–^COOH
1.0 bulk	0.00	0.00	0.00	0.00	1.00	0.0	0.00	0.00
1.0 surf.	0.00	0.00	0.00	0.12	0.76	0.09	0.03	0.00
6.0 bulk	0.00	0.00	0.00	0.00	0.00	1.00	0.00	0.00
6.0 surf.	0.00	0.26	0.07	0.19	0.19	0.24	0.02	0.02
9.5 bulk	0.00	0.28	0.08	0.00	0.00	0.08	0.57	0.00
9.5 surf.	0.06	0.32	0.18	0.12	0.12	0.06	0.09	0.06
12.5 bulk	0.00	0.00	1.00	0.00	0.00	0.00	0.00	0.00
12.5 surf.	0.30	0.21	0.34	0.05	0.07	0.00	0.02	0.02

a Data from ref (41).

b For each pH, species found at the surface but not in the bulk are marked red. The labels in the top row synthetically identify the different species through the protonation state at the three sites: amino, sulfur, and carboxylic.

The presence of molecules with charged groups at the surface can also affect the distribution of any cosolvated inorganic ions. In ref (14), we studied how the surface propensity of halides in the presence of selected amino acids depends on pH. The amino acid introduces “pH breaking points” at which the surface presence of halides increases. These pH breaking points correspond to the p*K*_a_ of the amino acid carboxylic and amine groups. For example, the surface presence of Cl^–^ increases significantly above the pH breaking points of phenylalanine. This behavior can be rationalized by realizing that phenylalanine has a high surface propensity due to its large hydrophobic side chain containing an aromatic ring (R = −CH_2_C_6_H_5_). Above the pH breaking points, its positively charged protonated group, NH_3_^+^, attracts negative Cl^–^ ions. This ion pairing increases the presence of Cl^–^ by anchoring these ions to the surface-enriched phenylalanine.

## Conclusions and Outlook

Liquid-jet XPS and theoretical calculations form a powerful combination for studying the surface of aqueous solutions, providing detailed information on how the surface composition differs from the bulk as a function of environmentally relevant parameters such as concentration and pH. We have shown how carboxylic acids and alkyl amines on their own exhibit a much higher surface propensity for the neutral than for the charged forms. Mixed together, the surface propensity is increased by cooperative ion-pairing effects. For amino acids, we have shown how the surface propensity is connected to the nature of the side group, and for cysteine, we observe a complex nonbulk-like speciation at the surface. The presence of molecular ions at the surface is also seen to affect the distribution of inorganic ions.

On a larger scale, the presence of molecules such as carboxylic acids, alkyl amines, and amino acids decreases the surface tension of cloud condensation nuclei and will also reduce the water vapor pressure required for droplet activation and thereby stimulate the growth of the particles. According to equilibrium thermodynamics, this can lead to vapor condensation and promote liquid cloud formation. Köhler’s formulation of the process combines the Kelvin effect, which describes the change in the saturation vapor pressure of water in terms of a curved surface, surface tension, and Raoult’s effect, which relates the saturation vapor pressure of water to the solutes.^42^ An important parameter in the formulation is the surface tension of the aerosol particle which is influenced not only by the curvature of the droplet but also by the concentration of the amphiphilic solutes. Dissolved molecules such as carboxylic acids, alkyl amines, and amino acids can thus decrease the surface tension due to their surface-active properties and lower the critical supersaturation for cloud droplet activation.

Results obtained by a combination of liquid-jet XPS and theoretical calculations thus provide a starting point for a microscopic understanding of atmospherically relevant surface properties and processes such as surface tension, condensation and evaporation rates, water accommodation, and the chemical aging of aerosols. Via aerosol growth and CCN activity, these microscopic surface phenomena have macroscopic effects on the radiative forcing. Our results demonstrate the importance of a detailed understanding of the surface composition of aqueous solutions, which is one of the key factors in improving the modeling of aerosols in climate models.
